# Functional magnetic resonance imaging providing the brain effect mechanism of acupuncture and moxibustion treatment for depression

**DOI:** 10.3389/fneur.2023.1151421

**Published:** 2023-03-21

**Authors:** Kelvin K. L. Wong, Jinping Xu, Cang Chen, Dhanjoo Ghista, Hong Zhao

**Affiliations:** ^1^The Research Center for Medical AI, Institute of Biomedical and Health Engineering, Shenzhen Institutes of Advanced Technology, Chinese Academy of Sciences, Shenzhen, China; ^2^Acupuncture and Moxibustion Department, Luohu District Hospital of Traditional Chinese Medicine, Shenzhen, China

**Keywords:** depression, functional magnetic resonance imaging, acupuncture, moxibustion, qi inner energy, brain functional network, brain activity

## Abstract

The efficacy of acupuncture and moxibustion in the treatment of depression has been fully recognized internationally. However, its central mechanism is still not developed into a unified standard, and it is generally believed that the central mechanism is regulation of the cortical striatum thalamic neural pathway of the limbic system. In recent years, some scholars have applied functional magnetic resonance imaging (fMRI) to study the central mechanism and the associated brain effects of acupuncture and moxibustion treatment for depression. This study reviews the acupuncture and moxibustion treatment of depression from two aspects: (1) fMRI study of the brain function related to the acupuncture treatment of depression: different acupuncture and moxibustion methods are summarized, the fMRI technique is elaborately explained, and the results of fMRI study of the effects of acupuncture are analyzed in detail, and (2) fMRI associated “brain functional network” effects of acupuncture and moxibustion on depression, including the effects on the hippocampus, the amygdala, the cingulate gyrus, the frontal lobe, the temporal lobe, and other brain regions. The study of the effects of acupuncture on brain imaging is not adequately developed and still needs further improvement and development. The brain function networks associated with the acupuncture treatment of depression have not yet been adequately developed to provide a scientific and standardized mechanism of the effects of acupuncture. For this purpose, this study analyzes in-depth the clinical studies on the treatment of anxiety and depression by acupuncture and moxibustion, by depicting how the employment of fMRI technology provides significant imaging changes in the brain regions. Therefore, the study also provides a reference for future clinical research on the treatment of anxiety and depression.

## 1. Introduction

Acupuncture is the essence of Chinese traditional medicine (CTM) and has a remarkable history of more than 2,000 years. Acupuncture is based on the theory that living beings have an inner energy, known as Qi, and it is the flow of this inner energy that sustains them. Acupuncture theory suggests that there are 12 main meridians through which Qi flows. These meridians correspond to the major internal organs of the body. For example, there is a liver meridian, a heart meridian, and other such organ meridians. In each of these meridians, Qi can become stagnant or deficient. Thus, according to the meridian theory, the stimulation of acupoints elicits functional responses with curative effects, which can be used to treat diseases in clinical practice. In this way, the practice of acupuncture has been recognized worldwide. Nevertheless, in today's era of medical practice based on medical technology, for the wider usage of acupuncture, there is a need to clarify its treatment mechanism by using modern medical technology.

Moxibustion is used concurrently with acupuncture in Chinese traditional medicine. Moxibustion is based on the concept that blockages in the flow of energy lead to mental and physical health problems. Moxibustion is a form of heat therapy in which dried plant materials called “moxa” are burned on or very near the surface of the skin. With the development of modern science and technology, medical imaging technology is extensively used to study the mechanisms of acupuncture and moxibustion based on brain imaging 82 especially functional magnetic resonance imaging (fMRI), positron emission scanning (PET), and single photon emission tomography imaging (SPECT).

Since the mid-1990s, modern science and technology has been increasingly employed to explore the physiological mechanisms underlying the curative effects of acupuncture and moxibustion. In this regard, fMRI is one of the most popular imaging technologies. fMRI can not only accurately and reliably locate specific cortical regions of brain activity (with spatial resolution up to several millimeters) but can also be used for repeatedly carrying out dynamic scanning to track changes in brain signals in real time (time resolution up to seconds) (1). It is noteworthy that fMRI has developed rapidly with numerous advantages such as non-invasive, non-radiation, high spatial resolution, and simultaneous functional and morphological imaging. At present, its application in the research study of the acupuncture mechanism within the brain organ regions is becoming more and more widespread.

Depression is a type of emotional disorder and a syndrome mainly characterized by low mood and a feeling of hopelessness (2). Relevant epidemiological studies have shown that the annual incidence of depression is ~0.8–9.6% worldwide, and 3–5% in China (3). At present, more than 26 million people have been affected by depression, and one-third of patients with depression have died from the disease. The incidence of depression is high, and the course of the disease is recurrent. In modern medicine, 5-HT reuptake inhibitors (SSRIs) are often used in the treatment of depression. However, a large number of clinical studies have reported that (i) long-term oral chemical prescription drugs can have debilitating anticholinergic side effects, resulting in poor patient compliance with the drugs and a high recurrence rate and (ii) on the contrary, acupuncture has good green clinical efficacy in the treatment of depression (4, 5).

In recent years, the study of the human brain using fMRI, involving cerebral blood flow velocity detection by analyzing the microbiota–gut-brain axis, has gained increasing attention in the study of psychiatric disorders. With the rapid development of fMRI technology, acupuncture therapy combined with fMRI technology can observe, explore, and study the central mechanism of acupuncture *in vivo* and non-invasively, thereby providing a modern visual research means for acupuncture treatment of depression (6). Acupuncture points are believed to stimulate the central nervous system, in turn, releasing chemicals into the muscles, spinal cord, and brain. These biochemical changes may stimulate the body's natural healing abilities and promote physical and emotional wellbeing. In this study, clinical research on the treatment of depression by acupuncture and moxibustion and fMRI technology-based imaging changes in the brain regions are discussed in detail, providing the basis for future clinical research on the treatment of depression.

## 2. Acupuncture and fMRI medical imaging modality techniques

### 2.1. Acupuncture methods

**Figure 1:**
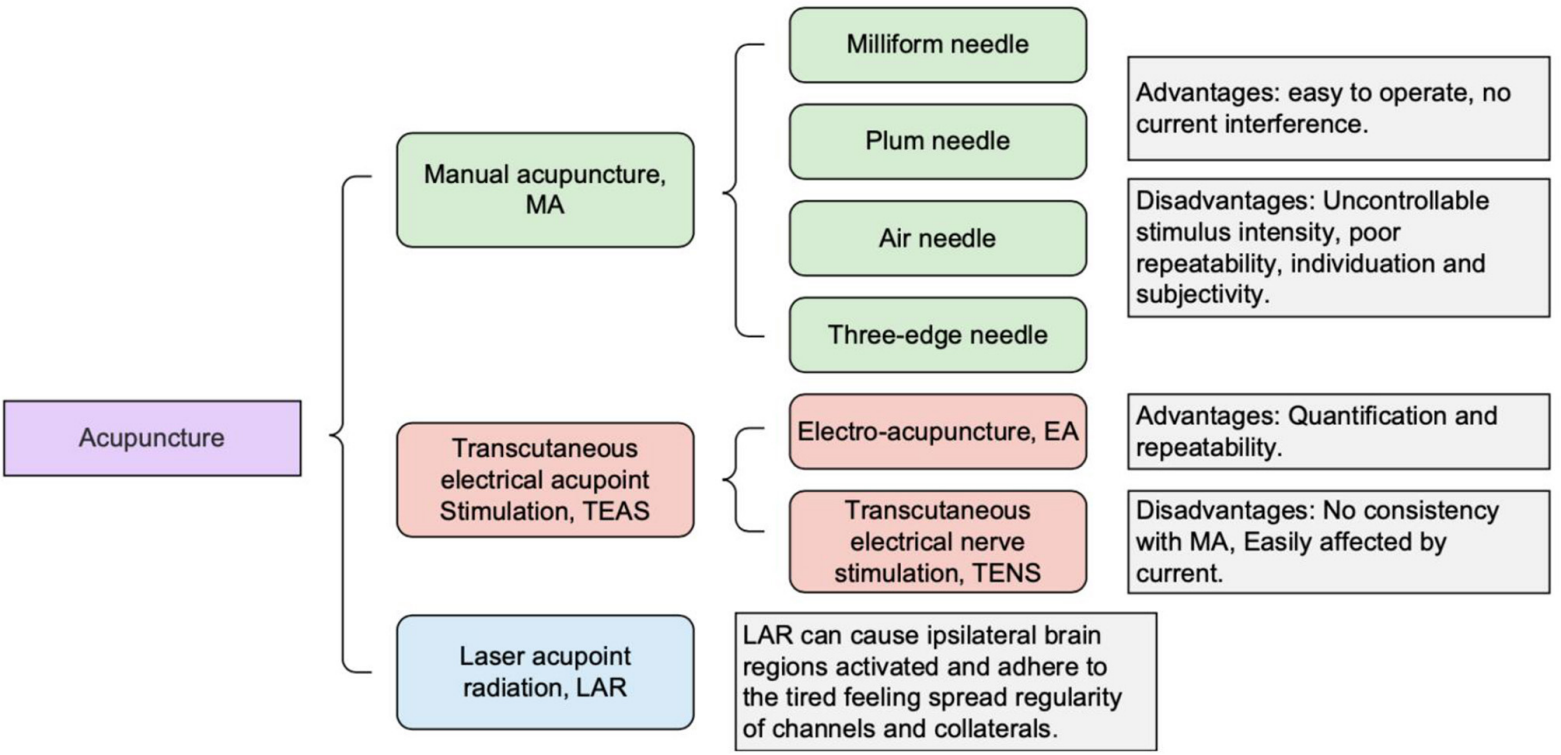
Summary of common acupuncture methods and devices employed.

The devices to study brain imaging of acupuncture and moxibustion methods mainly include (1) hand needle (manual acupuncture 82 MA), (2) percutaneous acupoint stimulation (transcutaneous electrical acupoint stimulation 82 TEAS), and (3) laser acupoint irradiation (also known as laser acupoint radiation 82 LAR). MA mainly employs the filiform needle and triangular and plum needle; the filiform needle is the most traditional acupuncture stimulation device. It is widely used to study the mechanism of the effect of acupuncture and moxibustion by fMRI. Its advantages are that it is simple, easy to operate, and there is no current interference; its disadvantages are that the stimulus intensity cannot be controlled, poor repeatability, and subjectivity. TEAS mainly includes electroacupuncture (EA) and percutaneous acupoint electric nerve stimulation (transcutaneous electrical nerve stimulation 82 TENS); its advantages (compared with the traditional MA) are quantified stimulation and repeatability; its disadvantages are that it is not completely consistent and fMRI imaging is easily affected by the current. Siedentopf et al. (1) have used LAR to irradiate the Xiaxi Acupoint (GB43), and the authors found that LAR can cause the activation of the corresponding brain region on the same side, abiding by the meridian sensory transmission law. These different methods of acupuncture and moxibustion to activate the brain, combined with fMRI brain imaging technology, provide the scientific basis for the further development of acupuncture to treat brain disorders associated with depression. A summary of the common acupuncture methods is provided in Figure 1.

#### 2.1.1. Balanced acupuncture and moxibustion (BAM)

Balanced acupuncture and moxibustion (BAM) is a modern acupuncture discipline that (1) inherits traditional medicine and incorporates modern scientific theories, (2) combines both the theory of mind regulation of traditional Chinese medicine and the theory of nervous regulation of Western medicine as the theoretical basis, and (3) focuses on the life gene program of brain science and involves exploring the law of development of life science, the law of evolution of disease science, and the law of self-intervention of repair science. This discipline has been established and has allowed the formation of an overall balanced medical regulation model of acupuncture and moxibustion adapted to psychology, physiology, nature, and society (7). This balanced acupuncture and moxibustion technology operates in the brain, the “core” of which is the cerebral nerve. In the treatment of brain disorders, such as depression, the positioning of BAM is in the brain.

The advantages of this balanced acupuncture and moxibustion (BAM) method include those as follows: (1) Patients are not selective: whether male or female and regardless of age (fetus, infant, infant, juvenile, youth, middle-aged, and elderly), as long as there are targeted symptoms, balanced acupuncture and moxibustion intervention can be carried out, and balanced acupuncture and moxibustion care can also be carried out on healthy people. (2) No disease limitations: Regardless of disease, BAM promotes brain upgrading and starts the patient's self-repair process by means of an acupuncture specific “life switch.” (3) Whether in internal medicine, gynecology, or pediatrics, balanced acupuncture intervention can be adopted. (4) No time restraints: Balanced acupuncture is not limited by time and can be performed at any time. (5) No conditions: The balanced acupuncture technique can be used for intervention as long as a silver needle is adopted. The clinical operation is simple and easy, and no special treatment environment and medical conditions are required, whether in the field of war or in peacetime, as well as on planes, ships, and trains, this balanced acupuncture intervention can be carried out at any time and any place. Especially for first aid at the scene of war, its role and value are even greater.

#### 2.1.2. Large meridian acupuncture techniques

In the so-called “big connection meridian,” (i) “big” refers to the 12 major meridians: five Yin meridians: the heart, spleen, lungs, kidneys, and liver; five Yang meridians: the small intestine, stomach, large intestine, urinary bladder, and gallbladder; the Pericardium meridian; and the San Jiao meridian and (ii) “connection meridian” refers to connecting the meridians. Connecting the meridians is achieved by connecting all 12 proper channels by needling the 12 Jing points so that Ying Wei Qi and blood in circulation can operate normally. In acupuncture, as a technique for balancing the flow of energy, the life force (known as Qi) is believed to flow through meridian pathways in the body. By inserting needles into specific points along these meridians, acupuncture balances the energy flow. The specific practice is to use 28 1-inch-long needles to acupuncture the original points and collaterals of the 12 meridians; in accordance with the sequence of meridians and collaterals, only pricking each point without leaving the needle inserted—one side each time, the other side the next time; prick for a total of four times for an old disease from the original point to the luo point, and for a new disease from the luo point to the original point. From the point of view of efficiency, stroke is the most suitable disease for this acupuncture method. Although there have been some problems recorded in previous studies on this acupuncture method, it can obviously improve the quality of life for patients, reduce the rate of stroke disability, reduce the economic burden of family and society, and provide significant social benefits. In addition to apoplexy, the spectrum of diseases cured by Dajiejing acupuncture is also gradually expanding, and its mechanism is gradually being clarified (8).

The following five points should be given attention in future clinical studies on the Dajiejing acupuncture technique: (1) Reasonable selection and clear expression should be made in the estimation of the number of cases to ensure the reliability of the conclusion; (2) the baseline homogeneity analysis should fully consider the prognostic factors of the diseases studied; (3) the scale of a clinical study should be in line with the different pathological characteristics of the patients studied at different stages; (4) the negative event report (safety evaluation) should be described truthfully; and (5) the influence of different acupuncture sequences, different stimulation means, and different acupoint selections on the technical efficacy of Dajiejing moxibustion remains to be further studied.

### 2.2. Techniques and methods of fMRI

**Figure 2:**
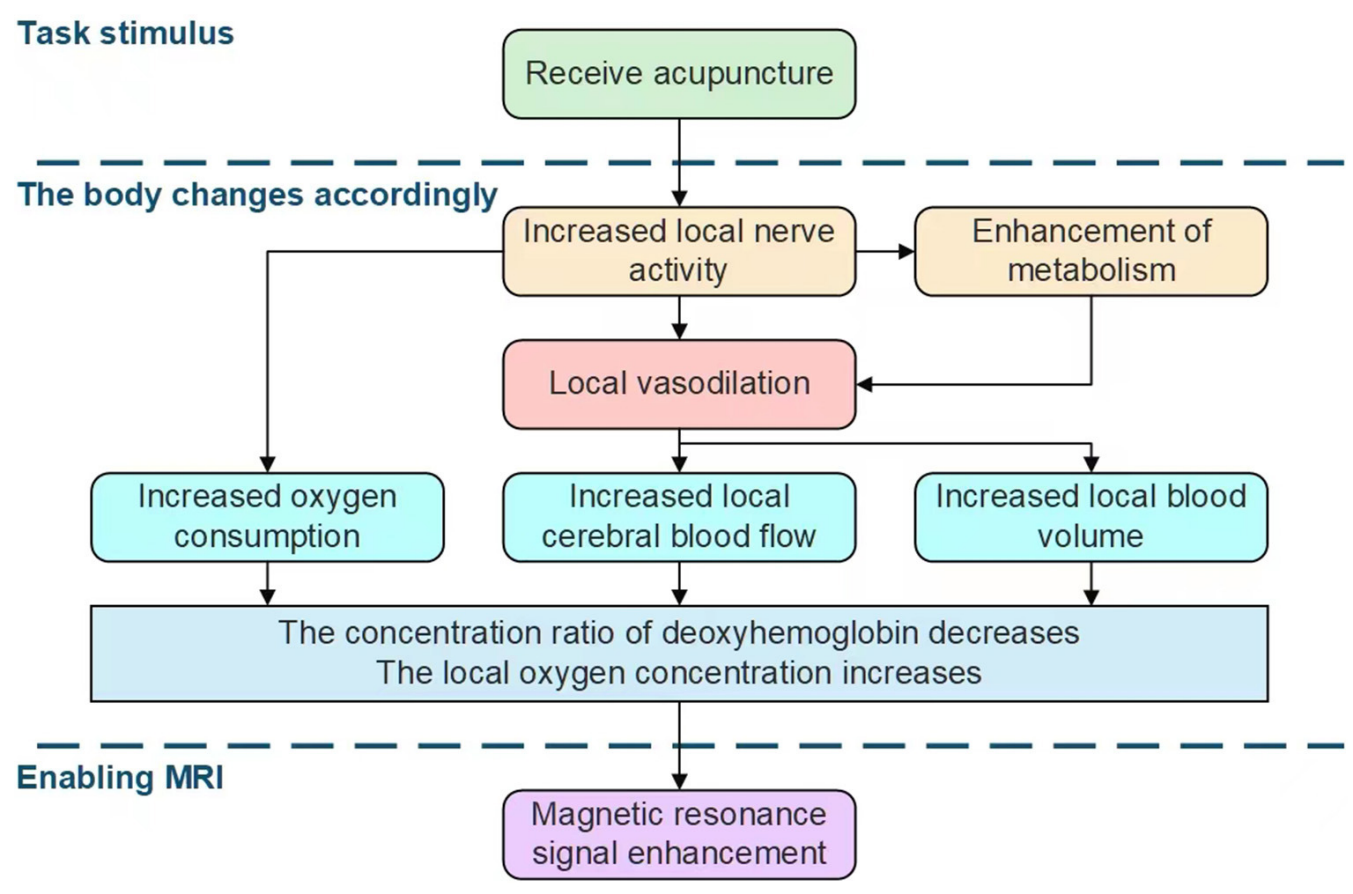
Introduction to the principles of fMRI and the effects of acupuncture on brain stimulation.

**Main principles:** As shown in Figure 2, in the initial stage of brain stimulation, the local brain activity is enhanced, oxygen consumption increases, and deoxyhemoglobin rises rapidly after the stimulation begins. Thereafter, due to the activation of brain function, local cerebral vascular dilation and increased blood flow result in a large amount of oxygen-rich blood flowing into the local area. Oxygen-rich blood increases rapidly *via* perfusion and the concentration of oxygen-rich hemoglobin in the local brain area rises rapidly, while deoxyhemoglobin begins to decline rapidly. The deoxyhemoglobin content eventually drops to the lowest value. With stimulation, the proportion of oxygenated hemoglobin is increased, while the proportion of deoxyhemoglobin is decreased, with both tending to an equilibrium state.

#### 2.2.1. Stimulation module design

The design of the stimulus module in fMRI mainly includes two categories that are based on the task state and resting state (9–11). The task state module design is the classic method of fMRI acupuncture research. In recent years, some studies have adopted design ideas that are more consistent with clinical models. Some studies have used the single block design or baseline unified processing method, but objectively, the single block design needs to be established as a mathematical model corresponding to its image processing in order to improve the reliability of its results. Considering these limitations of practical application, another design type based on the resting state has been widely used in fMRI research (9) and has been applied in the study of brain effects of acupuncture, which can partially replace the task mode module design. For example, the post-stimulus effect can be obtained from a comparison of the resting state before and after acupuncture. This method can simplify the design of the fMRI acupuncture stimulation module and can enable objective assessment of the long-term cumulative effects of acupuncture treatment so as to explore the mechanism of brain effects of acupuncture.

#### 2.2.2. Data post-processing methods

With the progress of modern medical imaging technology (9), data processing software for acupuncture fMRI research has developed a lot. The software packages SPM99, AFNI, FSL, and Freesurfer are commonly used, and they can also be obtained free of charge on relevant professional websites. A recently developed new method, “regional homogeneity” (12), has been gradually promoted and applied to the study of brain effects of acupuncture and moxibustion in China. It assumes that the neighboring voxels in a particular active brain region can be synchronized, but they are limited by functional connections that are spatially distant. Clinically, some confounding factors, such as respiration, heartbeat, and blood flow, require fMRI to extract signals of spontaneous nerve activity and to adopt reasonable data post-processing methods in order to accurately obtain and correctly interpret the test results.

#### 2.2.3. Real-time acupuncture and moxibustion fMRI study

As an emerging neuroimaging technology, fMRI has no radiation, strong repeatability, high accuracy, and good temporal and spatial resolution. It can be employed to (i) conduct real-time functional imaging of the brain tissue when acupuncture induces brain activity, (ii) study functional connections of brain regions after acupuncture operation, and (iii) observe the influence of acupuncture acupoints on the default brain network. It can provide objective and visual real-time images and theoretical interpretation for acupuncture treatment of diseases. The main obstacle to the visualization of the real-time brain effects of acupuncture lies in the difficulty of implementing acupuncture points on the head. In addition, when the electroacupuncture stimulator is placed in the MRI magnet room for close adjustment operation, its current can interfere with the MRI signal and affect the image quality of real-time fMRI. Resolving these obstacles depends on the design of the head coil and the development of anti-magnetic field interference electroacupuncture technology.

Real-time fMRI monitoring has been used to study the effects of acupuncture on the brains of patients with Parkinson's disease, and increased activation was found in the cingulate gyrus and the cerebellum (13). In the study of acupuncture treatment for heroin addicts, it was found that there was significant activation in the thalamus (14). In the study of children with spastic cerebral palsy, acupuncture and moxibustion more frequently induced a negative activation of the primary motor cortex, parahippocampal gyrus, and higher cognitive brain areas, as well as activation of the cuneus and the insula (15).

### 2.3. Study of the relationship between different acupuncture methods and their brain effects

fMRI can not only monitor brain functional activities in living tissues in real time and dynamically but can also observe the activation of different brain functional regions by different acupuncture methods (16), thereby opening up a new way for the study of the acupuncture mechanism.

#### 2.3.1. Hand acupuncture stimulation

Hui et al. (17) proposed the theory that hand acupuncture modulated the “limbic lobe-parlimbic [*sic*] lobe-neocortex network,” postulating that this network plays an important role in human cognition, emotion, memory regulation, and internal environmental stability. This has provided a new scientific record of the brain effect mechanism of acupuncture, and it has laid out a theoretical foundation for the treatment of some mental diseases such as depression by hand acupuncture. The depth of acupuncture with different hand needles has shown significant differences in the stimulus-induced brain function regions. Deep acupuncture showed more negative activation in the limbic system, whereas shallow acupuncture showed more activation in somatosensory, motor, and language areas (Broca and Wernicke areas) (18). The duration of hand acupuncture also has stimulating effects on the different functional areas of the brain. The somatosensory area, limbic system, visual area, language area, or higher cognitive area are better activated by hand needle rotation compared with hand needle retention (19, 20). Longer stimulation time is more likely to induce (i) activation of the lower frontal lobe, parietal, occipital, cerebellum, or temporal poles and (ii) negative activation of the prefrontal cortex, orbital gyrus, or pontine activation. Table 1 depicts the results of hand acupuncture stimulation under different conditions.

**Table 1:** The results of hand acupuncture stimulation under different conditions.

**Different conditions**	**Emergence results**
Deep acupuncture into the limbic system	No activation
Deep acupuncture into the somatosensory area	Activation
Short stimulation time	Activation of somatosensory area, limbic system, and so on
Long stimulation time	Activation in the lower frontal lobe, cerebellum, and negative activation

#### 2.3.2. Electroacupuncture stimulation

Fang et al. (9) and Hui et al. (17) adopted a new method of short-range and long-range connection of the brain functional network to analyze the brain functional states during and after needle retention in electroacupuncture, and found dynamic changes in brain function. The authors proposed the hypothesis that electroacupuncture stimulation of the acupoints can activate the “limbic lobe-prefrontal network.” This hypothesis opens up a new way to treat diseases by stimulating the functional regions of the brain with electroacupuncture.

Electroacupuncture can induce (i) more activation in the somatosensory area, motor area, brainstem, cingulate gyrus, and insula and (ii) more negative activation in the septum or precuneus (18, 21). Napadow et al. (21) found that 2 Hz stimulation was more likely to activate the brainstem than 100 Hz stimulation. However, Li et al. (22) found no statistical difference in the effects between 2 and 20 Hz stimulations. Electroacupuncture stimulation positively and negatively activates different functional areas of the brain; however, whether the activation of different functional areas of the brain is affected by the intensity of electroacupuncture stimulation with different frequencies needs to be further studied and verified.

#### 2.3.3. Fake acupuncture point stimulation

In domestic studies, sham acupoints (adjacent or distant non-meridian acupoints) were used as controls, while in foreign countries, sham stimulation methods (such as Streitberger acupuncture or acupoint skin VonFrey ciliary mechanical stimulation acupuncture, also known as comfort acupuncture) were used as controls.

Schockert et al. (23) found that acupuncture could induce more activation of brain motor areas in patients with stroke. Li et al. (24) found that sham acupuncture (brush stimulation of the skin) could activate more somatosensory and motor areas in patients with stroke. Napadow et al. (25) compared the effects of hand acupuncture on patients with carpal tunnel syndrome and on healthy volunteers and found that hand acupuncture induced a greater activation of the lateral hypothalamic region in patients. Sham acupuncture also induced greater activation in somatosensory, cognitive, and emotional brain regions. Kong et al. (26–28) proposed that acupuncture for pain relief was different from, but obviously influenced by the placebo effect, and the placebo effect was different between the real point and the fake point. These studies on the comparison of true and false acupuncture point stimulation in patients showed that there were significant differences between brain function regions activated by false acupuncture point stimulation and true acupuncture point stimulation, which needs to be confirmed by further studies.

### 2.4. fMRI study on different acupuncture acupoints and stimulation areas

#### 2.4.1. Activation and negative activation

In recent years, in fMRI studies on the brain effects of acupuncture and moxibustion, the negative activation effect has become the focus of attention, but its mechanism is still unknown. Whether it is related to the inhibition of the function of neural nuclei needs to be further studied in terms of neurotransmission or electrophysiology. The literature has summarized the results of brain functional imaging studies on 18 acupoints along nine meridians and it was found that multiple acupoints distributed along the same meridians induced similar patterns of brain activation and negative activation. For example, acupoints distributed along the stomach meridian showed the activation of the superior limbic gyrus and negative activation in the posterior cingulate gyrus, hippocampus, and parahippocampal regions (11). Fang et al. (9) found that electroacupuncture stimulation of the visceral area of the ear (the auricular vagus nerve area) could produce a significant negative activation effect on the nucleus of the solitary tract of the brain stem, nucleus locusuleus, and marginal lobe. Other research studies showed that acupuncture point stimulation could induce the activation of the first synesthesia area, the second synesthesia area, the thalamus, and the insula. The main brain area of the limbic lobe system was mostly negatively activated, and the brain stem and cerebellar vermis appeared to be either negatively activated or showed no activation phenomenon. These results provide a clear understanding and application of the mechanism of the brain function effect of acupuncture and moxibustion in the treatment of diseases.

#### 2.4.2. The specificity of activation intensity and the activation region

Some studies showed that electroacupuncture at Zusanli and Guanyuan points has a common feature, both of which can induce (i) a significant negative activation of the limbic lobe-neocortex (anterior inferior cingulate gyrus-medial frontal gyrus) pathway as well as (ii) the enhancement of short-range connections in this brain functional area (29). Acupuncture at the Zusanli point shows a stronger brain effect, which reflects the relative specificity of the acupoint. Several domestic and foreign studies on fMRI monitoring of the brain effect of acupuncture, especially through the comparison of fMRI studies on major acupuncture points (such as Hegu, Zusanli, and Taichong), have shown that there was little difference in the brain effect between different acupuncture points, and that there was more overlap in the activation area and activation intensity. Electroacupuncture at Dadun and Yinbai points produced negative activation effects on the ipsilateral somatosensory area and insula lobe, but no activation was observed in the marginal lobe. Simple heat pain stimulation showed that the somatosensory II region of the thalamus and the insula of the anterior cingulate cortex were activated. Pure electroacupuncture stimulation showed a negative activation of the contralateral raphe magnus, inferior frontal gyrus, and amygdala (30). The brain effects of skin electrical stimulation at Zusanli and Sanyi acupoints on normal people in cold pain showed that it (i) positively activated the bilateral somatosensory area II, medial prefrontal gyrus, and cingulate gyrus, and (ii) negatively activated the contralateral somatosensory area, parietal cuneus, and middle cingulate gyrus (31).

### 2.5. Changes of functional nuclei and brain areas under fMRI related to depression treated by acupuncture

**Figure 3:**
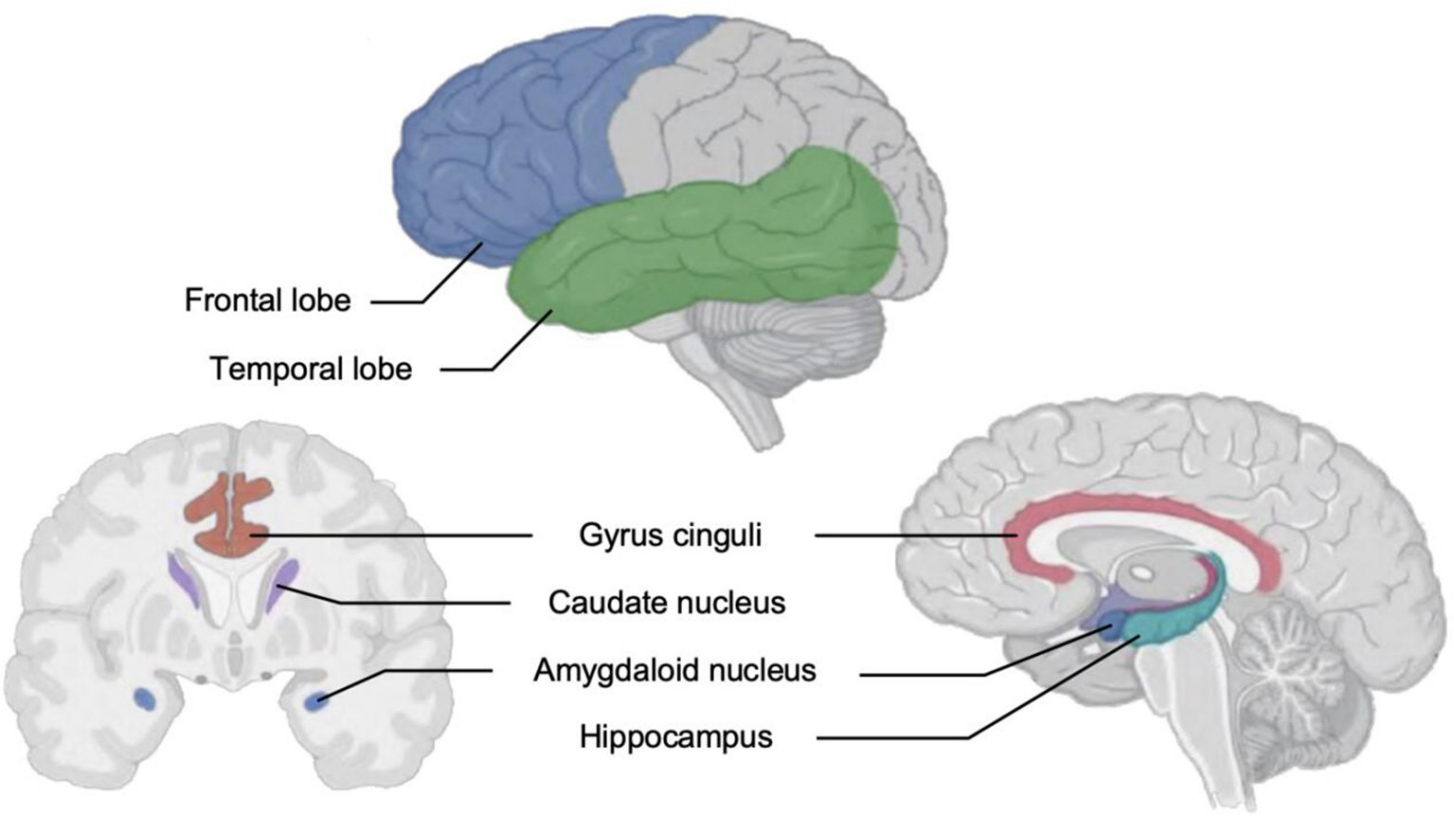
Brain regions associated with depression that changed with acupuncture intervention.

Existing studies (32) have shown that changes in various indicators in different brain areas, such as the prefrontal cortex, cingulate cortex, hippocampus, striatum, amygdala, and thalamus, may mediate anxiety and depression. In resting-state fMRI, it can be observed that the changes in the above parts of nuclear masses and brain regions are significant in people with depression, and acupuncture has a distinct influence on the changes of related nuclear masses and brain regions (33). The brain regions associated with depression that changed after acupuncture intervention are shown in Figure 3.

**Figure 4:**
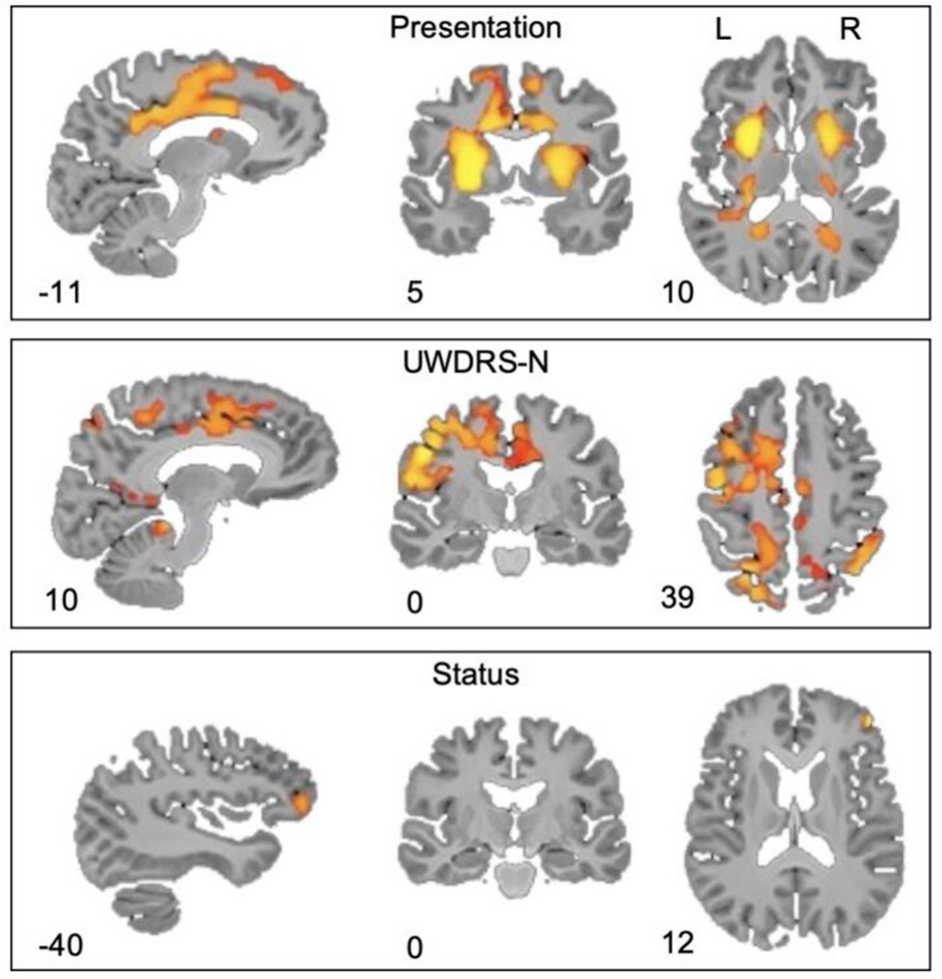
Quantitative MRI of the whole brain. The absolute magnetic sensitivity of patients with neurological symptoms was significantly higher than that of patients with liver symptoms, based on the increase of the UWDRS-N score; the magnetic sensitivity of patients with active symptoms was higher than that of patients with stable symptoms, with a FEW-corrected *p*-value.

Wilson's disease is an autosomal recessive disorder of copper metabolism, associated with neurological and hepatic abnormalities. Chelation therapy is used to flush copper, but the neurological symptoms remain uncontrollable. Neuroimaging studies have uncovered a range of abnormal outcomes, have increased understanding of the disease mechanisms, and have provided avenues for the study of the disease prognosis and biomarkers that can be used for detection. Whole brain quantitative magnetic susceptibility mapping analysis revealed iron deposits in the putamen, cingulate gyrus, and medial frontal cortex in patients with neurological manifestations. In the treatment of chronically affected patients, the severity of neurological symptoms is associated with extensive cortical iron deposits (Figure 4).

#### 2.5.1. Cingulate gyrus

As a cortical part of the limbic system, the cingulate gyrus is located between the cingulate sulci and the corpus callosal sulcus, the medial side of the cerebral hemisphere. It is mainly responsible for the integration of emotional information and attention and plays a role in evaluation and execution (34). The anterior cingulate gyrus (ACC) has extensive fibrous connections with many cortical and subcortical structures, and is involved in the regulation of emotion, mood, and other functions. When receiving negative emotional stimuli, the ACC can be observed to be significantly activated, and so ACC is regarded as a key structure in the pathogenesis of patients with depression (35). fMRI results showed that, compared with healthy volunteers, patients with depression exhibited reduced whole brain connectivity of several regions such as the medial and lateral prefrontal cortex (36), and enhanced functional connectivity between the anterior dorsolateral anterior cingulate cortex and the dorsolateral frontal lobe (37).

Existing studies (33) have shown that electroacupuncture at the Baihui point can (i) regulate the default mode network in patients with depression and (ii) induce enhanced functional connectivity between the posterior central gyrus, the prefrontal cortex, and the bilateral ACC in patients with depression. Some studies (38) showed that the values of ALFF (amplitude of low-frequency fluctuations) of the left ACC/left medial orbital superior frontal gyrus and the left middle occipital gyrus/left angular gyrus decreased in patients with recurrent depression after auricular nail acupuncture treatment. Acupuncture treatment can improve both intestinal and psychiatric symptoms in patients with diarrheal irritable bowel syndrome and the associated mechanism may be that acupuncture regulates the limbic system-neocortex-related brain regions to restore balance (39). Studies (40) have shown that both electroacupuncture and moxibustion can improve symptoms and relieve depression in patients with constipation-type irritable bowel syndrome, with electroacupuncture having a more significant effect than moxibustion; furthermore, it was observed by fMRI that the ALFF values of the ACC, insular cortex, and prefrontal cortex in patients decreased after treatment.

#### 2.5.2. Amygdala

The amygdala is an important subcortical nucleus mass in the limbic system of the brain, which is involved in a variety of emotional processes. By exploring the differences in ALFF among the subregions of the amygdala, it has been concluded that the abnormal function of the left amygdala may be the neuropathological basis in patients with depression (41). Some works (42) have studied the correlation between anxiety and depression by employing fMRI to observe the neural matrix of anxiety and depression and its interaction effect on the functional connectivity network of the amygdala in patients with depression: the study results showed that the amygdala connectivity strength mediated the relationship between anxiety and depression in patients with depression. The degree of anxiety and depression in patients with Crohn's disease is related to the functional connection between the amygdala and the thalamus (43). In some studies (44), patients with depression were treated by stimulating the transcutaneous vagus nerve (tVNS), and their Hamilton depression scale scores were significantly reduced. fMRI results showed that tVNS could significantly regulate the resting-state functional connectivity of the lateral amygdala forehead in patients with depression.

Electroacupuncture can induce (i) a decrease in functional connections between the amygdala and the orbitofrontal cortex in patients with depression and (ii) an increase in functional connections between the amygdala and the hippocampus, precuneus, anterior central gyrus, and angular gyrus in patients with depression, thus changing the abnormal amygdala network connections in patients with depression (45). Acupuncture at the Hegu and Zusanli points can activate the hypothalamus and the nucleus accumbens and reduce the excitability of the pretuberous cortex, amygdala, and hippocampus; it can also activate the structure of the down-pass analgesic pathway and inactivate multiple limbic brain regions related to pain so as to achieve an analgesic effect and relieve patients' depression (46).

#### 2.5.3. Hippocampus

Located between the brain's thalamus and the medial temporal lobe, the hippocampus is part of the limbic system, and it is responsible for functions such as storage conversion and orientation of long-term memory. Studies (47) have shown that, compared with healthy subjects, patients with anxiety disorders had: (1) lower ALFF values in the right posterior central gyrus and right anterior central gyrus, (2) lower ReHo values in the right anterior central gyrus, right posterior central gyrus, and left anterior central gyrus, and (3) higher ReHo values in the left posterior cingulate cortex. The ALFF value of the left hippocampus was higher in subjects with symptom remission, and the ReHo value of the right posterior central gyrus was higher in subjects without symptom remission. Electroacupuncture at the Baihui point can reduce the functional connections between the posterior central gyrus/prefrontal cortex and the left middle prefrontal cortex as well as between the left angular gyrus and the bilateral hippocampus/accessory hippocampus in patients with depression (33).

Existing studies (48) have also shown that the functional connection between the hippocampus and ventral cephalic putamen in patients with depression under resting-state fMRI was significantly enhanced during acupuncture intervention. Some studies (49) have been conducted on patients with post-traumatic stress disorder accompanied by anxiety and depression, and the results showed that the patients' symptoms improved after electroacupuncture treatment. fMRI showed that (i) functional connections were enhanced in the bilateral hippocampus, right posterior central gyrus, and left parahippocampal lobule and (ii) connections between the left parahippocampal gyrus and the right amygdala were inhibited. Therefore, it can be inferred that the improvement in anxiety and depression symptoms in post-traumatic stress disorder by electroacupuncture may be related to (i) the enhancement of connectivity between the parietal lobe and the hippocampus, and (ii) the inhibition of connectivity between the hippocampus, parahippocampal gyrus, and amygdala, leading to indirect effects on the limb system.

#### 2.5.4. Dorsal raphe nucleus

The dorsal raphe nucleus is a heterogeneous brainstem nucleus located in the ventral part of the midbrain aqueduct between the upper end of the pontine and the upper and lower colliculus of the midbrain. Its neurons use a variety of transmitters to control a variety of physiological functions, including learning, memory, and emotion, and it is closely associated with brain dysfunction (50). This whole nucleus mass is roughly divided into three parts: the ventral part, the lateral part, and the dorsal part (51). The dorsal raphe nucleus contains a large number of serotonergic neuron cells (52), which are closely related to anxiety (53). Research (54) has shown that the synthesis and release of serotonin neurotransmitter in the dorsal raphe nucleus can be regulated by electroacupuncture intervention for pain and depression. Anand et al. (55) analyzed depressed adolescents who did not use drugs. Resting-state fMRI observation showed that these subjects had abnormalities in the dorsal raphe nucleus-forehead and dorsal raphe nucleus-cingulate gyrus connection, and the severity of their depressive symptoms was related to the dorsal raphe nucleus-amygdala/hippocampus connection.

#### 2.5.5. Frontal lobe

The frontal lobe is the mind control center of the brain, and it is strongly associated with changes in cognitive functions, such as task execution, memory retrieval, information integration, and emotional regulation in patients with depression. Studies (56, 57) have shown that patients with depression have significantly abnormal neural activity in the frontal lobe, the temporal lobe, and the circuitry between them. In the face of negative emotions, both cognitively prone patients with depression and patients with depression showed a significantly reduced activation of the dorsolateral prefrontal lobe and a significantly enhanced activation of the amygdala under fMRI, and the functional connection between the amygdala and the prefrontal lobe decreased. This indicated persistent state could become a high-risk factor for other diseases (58). Studies (59) have shown weakening of the functional connections between the left anterior cingulate gyrus and the left prefrontal cortex under fMRI in persons with bipolar depression (58). Some studies (60) have even used fMRI to show that acupuncture at the Taichong point can inhibit bilateral frontal lobes and most temporal lobes in patients with depression, and the change of the frontal lobe is likely to be one of the mechanisms of acupuncture in treating depression. After acupuncture treatment, the brain function of the prefrontal limbic region in patients with depression showed changes, and the functional connection between the right ventrocephalic putamen and the right dorsolateral prefrontal cortex as well as that between the bilateral cerebellar tonsil and the right dorsolateral caudate nucleus was reduced; thus, it was inferred that acupuncture could achieve therapeutic effects by regulating the prefrontal limbic region and the reward/excitation circuit of the striatum in patients with depression (48).

#### 2.5.6. Temporal lobe

The anterior part of the temporal lobe is the mental cortex, which (i) is closely related to emotion, involved in emotion, memory, hearing, and speech processes and (ii) performs the visual integration function of classifying pictures and objects. Some studies (61) have shown that the ReHo value of the right medial temporal gyrus and bilateral fusiform gyrus increased in patients with mild to moderate depression. Some studies (62) have also shown that (i) the functional connections between the left fusiform gyrus, right middle temporal gyrus, left inferior temporal gyrus, and medial prefrontal cortex were enhanced in patients with depression and (ii) abnormalities of the temporal lobe were associated with memory decline and emotional symptoms in patients with depression. Compared with the control group, the functional connections between the ventral inferior striatum and the medial prefrontal cortex, between the ventral putamen and the amygdala/hippocampus, and between the dorsal caudal gyrus and the middle temporal gyrus were significantly enhanced in patients with depression after acupuncture treatment. The ALFF values of the left putamen, globus pallidum, and caudate in patients with primary insomnia were lower than those of healthy subjects, while the ALFF values of the bilateral anterior central gyrus, bilateral posterior central gyrus, left cuneus, superior frontal gyrus, middle frontal gyrus, and middle temporal gyrus were higher in patients with primary insomnia. The ALFF values of the left putamen, globus pallidum, and caudate increased after treatment, while the ALFF values of the left precuneus, superior frontal gyrus, and middle temporal gyrus decreased (63).

## 3. Current status of the brain functional network based on fMRI, providing the mechanism of acupuncture treatment of depression

Acupuncture and moxibustion have become the main intervention measures for the treatment of depression because of their convenient operation, simple technique, obvious effect, and low price. However, the mechanism of the central effect of acupuncture and moxibustion on depression has not been scientifically explained. The emergence of fMRI technology provides an advanced means to explore the central mechanism of acupuncture and moxibustion through the brain functional network of people with depression. In recent years, many researchers have proposed a new discipline of “acupuncture imaging” (64, 65). The application of fMRI technology to study the mechanism of acupuncture treatment has become a hot topic in today's acupuncture and moxibustion classes. As far as the current research is concerned, it is generally believed that the default network in the functional network of the resting brain is closely related to the treatment of depression by acupuncture, which may be realized through the mutual integration of acupuncture information and depression information in the brain; however, this still needs to be further explored.

Wang et al. (66) treated patients with depression for 8 weeks using the acupuncture method of smoothing the liver and strengthening the spleen, and the results showed that the resting-state fMRI data had significant changes, and that regulation of the thalamic-cingulate gyrus-parahippocampal gyrus-frontal neural pathway was the basis for the regulation of neural information in the brain in the treatment of depression by acupuncture. Ananda et al. (67) applied fMRI to study the cerebral cortex margin and found that functional connections of the amygdala, striatum, cingulate gyrus, and medial thalamus were significantly weakened in patients with depression. The results showed that the anterior cingulate gyrus' regulation function on the limbic region was weakened in patients with depression, resulting in abnormal emotional regulation. Table 2 summarizes the research methods and findings of different research groups on acupuncture treatment for depression.

**Table 2:** Research status of the central mechanism of acupuncture treatment for depression.

**Research group**	**Research method**	**Research finding**
Wang et al. (66)	The treatment of depression using the acupuncture method of smoothing the liver and strengthening the spleen.	The modulation of thalamic-cingulate gyrus-parahippocampal gyrus-frontal neural pathway is the basis of neural information regulation in the treatment of depression using acupuncture
Ananda et al. (67)	The limbic cortex was studied using fMRI.	Patients with depression presented weakened anterior cingulate gyrus to limbic zone regulation function, resulting in abnormal emotional regulation.
Qu et al. (68)	Electroacupuncture of the Yintang and Baihui points for treating mild and moderate primary depression.	The ALFF of the right precuneus and middle frontal gyrus increased significantly, the ReHo value of the right middle frontal gyrus and left middle temporal gyrus increased significantly, and the ReHo value of the right caudate nucleus decreased significantly.
Institute of Acupuncture and Moxibology of China Academy of Chinese Medical Sciences (69)	A new concept of “neuroregulatory technology in brain science” is proposed.	Stimulation through the nucleus tractus solitarius-limbic lobe-brain default network is the key to ear acupuncture therapy.

Qu et al. (68) used electroacupuncture at the Yinang and Baihui points for the treatment of mild and moderate primary depression, with fMRI used to determine the resting-state ALFF data and ReHo data of patients with depression. After acupuncture, the ALFF of the right precuneus and middle frontal gyrus increased significantly, the ReHo value of the right middle frontal gyrus and left middle temporal gyrus increased significantly, and the ReHo value of the right caudate nucleus decreased significantly. In addition, the Institute of Acupuncture and Moxibology of China Academy of Chinese Medical Sciences also put forward a new concept of “neuroregulation technology of brain science” (69) and used fMRI to preliminarily explain the mechanism of electroacupuncture stimulation of the visceral region of the ear thyroid in the treatment of depression. The institute found that stimulation through the nucleus of the solitary tract-limbic lobule-brain default network is the key to ear acupuncture treatment.

Acupuncture and moxibustion in the treatment of depression relieve depression and soothe the patient, which can promote emotional stability and peace of mind both physically and psychologically. On the one hand, in the treatment of depression, the brain's functional network of acupuncture and moxibustion plays a potential regulatory role. On the other hand, the objective proof of fMRI provides an effective verification of the brain effect mechanism of acupuncture and moxibustion in the treatment of depression. However, the current research is limited to discussion of a single brain functional network, and there is a lack of integrated studies on depression-related brain functional networks. There are still many unexplored brain functional networks related to depression. Therefore, future studies can explore the integration and regulation effects of acupuncture on depression-related brain functional networks.

## 4. Discussion

fMRI has the advantages of being non-invasive, immediate, and providing accurate measurement of the brain response during acupuncture. It has helped to make some progress in the field of acupuncture science, but there are still some problems in the current research results. The research results of acupuncture are affected by many factors, such as the needle apparatus, acupuncture operation method, and degree of stimulation. At the same time, fMRI is also affected by the human body state, psychological factors, and visual and auditory conditions. In addition, there are some problems with fMRI itself, such as image quality and low temporal resolution. With the continuous development of fMRI imaging and its image processing technology, a broader approach can be created for (i) the study of the acupuncture mechanism, (ii) further improvement of research methods, experimental design, and image processing methods, (iii) strict control of interference factors, and (iv) disease-centering combined with acupuncture technology, supplemented by EEG, PET-CT, and other technologies. Combining the results of brain functional imaging with neuroanatomy, neurobiology, and neuroelectro-physiology, to conduct multidisciplinary, multi-level, and multi-system research, can help to further clarify the therapeutic mechanism of acupuncture and finally guide the clinical practice of acupuncture with the results of fMRI research to provide future structuring of this field.

“Resting-state fMRI” enables the development of data for neuronal activity and neuronal synchronization in the brain, and it has been applied to a certain extent in the study of the central mechanism of acupuncture in the treatment of depression. The development of “resting-state fMRI” provides the basis for an objective evaluation of the clinical efficacy of research on anxiety and depression and greatly promotes the development of the “brain function mechanism” of acupuncture stimulation at acupoints. However, there are still some problems in the related research, such as patients' fear of acupuncture treatment, the claustrophobia of fMRI, and the influence of different acupuncture techniques and acupuncture operators on the results of fMRI during acupuncture operations. If this “resting-state fMRI” study can be combined with various functional brain imaging techniques (such as electroencephalography), as the basis of controlling irrelevant variables, then the credibility of the research results can be increased. In the future, how to design experiments more reasonably, how to solve the problem of quantitative stimulation, and how to better apply fMRI technology to acupuncture research may become the direction of researcher efforts.

As a non-drug alternative therapy, according to statistics, acupuncture and moxibustion treatment has a good effect on more than 100 diseases (70). In recent years, fMRI technology has been gradually applied in the study of the brain effect mechanism of acupuncture for depression, functional dyspepsia, facial palsy, stroke, and other diseases, and it has made phased progress in clinical practice.

Chau et al. (71) observed the condition of patients with aphasia after 8 weeks of treatment after a stroke. The authors found that the change in the degree of aphasia was related to the activation of Wernicke's language area, and speculated that acupuncture treatment may be beneficial to the recovery of aphasia. Studies have found that the application of fMRI scanning can accurately locate the epileptogenic focus of patients with epilepsy, so as to avoid surgical damage to the surrounding normal brain function area and reduce postoperative brain dysfunction (72). In the study of acupuncture analgesia, acupuncture stimulation can inhibit the pain-activated brain network under the condition of Qi and transform the pain-activated brain area into an inhibited state to thereby achieve the analgesic effect of acupuncture (73). Studies have shown that electroacupuncture stimulation of the auricular thyroid vagus nerve can effectively treat mild to moderate depression in clinical practice, and its mechanism is regulation of the functional state of the vagus nerve, so as to change the emotion control center in the brain. Other studies have found that acupuncture at the Hegu point can (i) simultaneously activate the hand projection area and also the facial and oral projection area of the primary sensory cortex of the central posterior gyrus and (ii) activate the motor cortex of facial and oral regions, which directly reflects the close connection between the Hegu point and facial and oral regions (74, 75), thereby providing objective evidence for the theory of “facial and oral Hegu.” Studies have shown that the brain mechanism of acupuncture in the treatment of functional dyspepsia may be related to the fact that acupuncture significantly regulates the homeostasis network of the internal environment and important functional brain areas in the cerebral intestinal axis, including the insula, anterior cingulate cortex, and hypothalamus (76–79). Table 3 depicts the clinical progress on the central mechanism of acupuncture and moxibustion studied by fMRI.

**Table 3:** Clinical progress in the central mechanism of acupuncture and moxibustion studied by fMRI.

**Research group**	**Clinical progress**
Chau et al. (71)	The change of aphasia degree in patients with aphasia after stroke is related to the activation of Wernicke's language area. It is speculated that acupuncture treatment may be beneficial to the recovery of aphasia.
Wang et al. (72)	Accurate location the epileptic foci in patients with epilepsy, so as to avoid surgical damage to the surrounding normal brain function area and reduce postoperative brain dysfunction.
Fang et al. (73)	Acupuncture stimulation can inhibit the pain brain network activated by pain, so as to achieve the analgesic effect of acupuncture.
Li et al. (74)	Electroacupuncture stimulation of the auricular vagus nerve can effectively treat mild and moderate depression in a clinical setting.
Li et al. (75), Wu et al. (76)	Acupuncture at the Hegu point can simultaneously activate the hand projection area and the facial and oral projection area of the primary sensory cortex of the central posterior gyrus and activate the motor cortex of the facial and oral cortex.
Zeng et al. (77), Fang et al. (78)	The cerebral mechanism of acupuncture in the treatment of functional dyspepsia may be related to the significant regulation of acupuncture in the homeostasis network of the internal environment and important functional brain regions in the cerebral intestinal axis, including the insula, anterior cingulate cortex, and hypothalamus.

## 5. Limitations of acupuncture effects observed using fMRI

(1) Studies using fMRI to observe the brain regions activated by acupuncture effects are limited to a few acupoints, and the subjects are mostly healthy people; thus, few clear conclusions have been drawn.
(2) fMRI indirectly reflects blood oxygen saturation and blood flow through the measurement of the MR signal, but it cannot directly show the functional activities of nerve cells.
(3) There is no set of scientific standard experimental designs when conducting acupuncture experimental research; this may be the reason why many similar experiments reach different conclusions, which needs to be solved urgently.
(4) The feeling of “getting Qi” in the process of acupuncture and moxibustion is the subject's subjective feeling, and there is no objective standard, which will also have a certain impact on the results.

**Figure 5:**
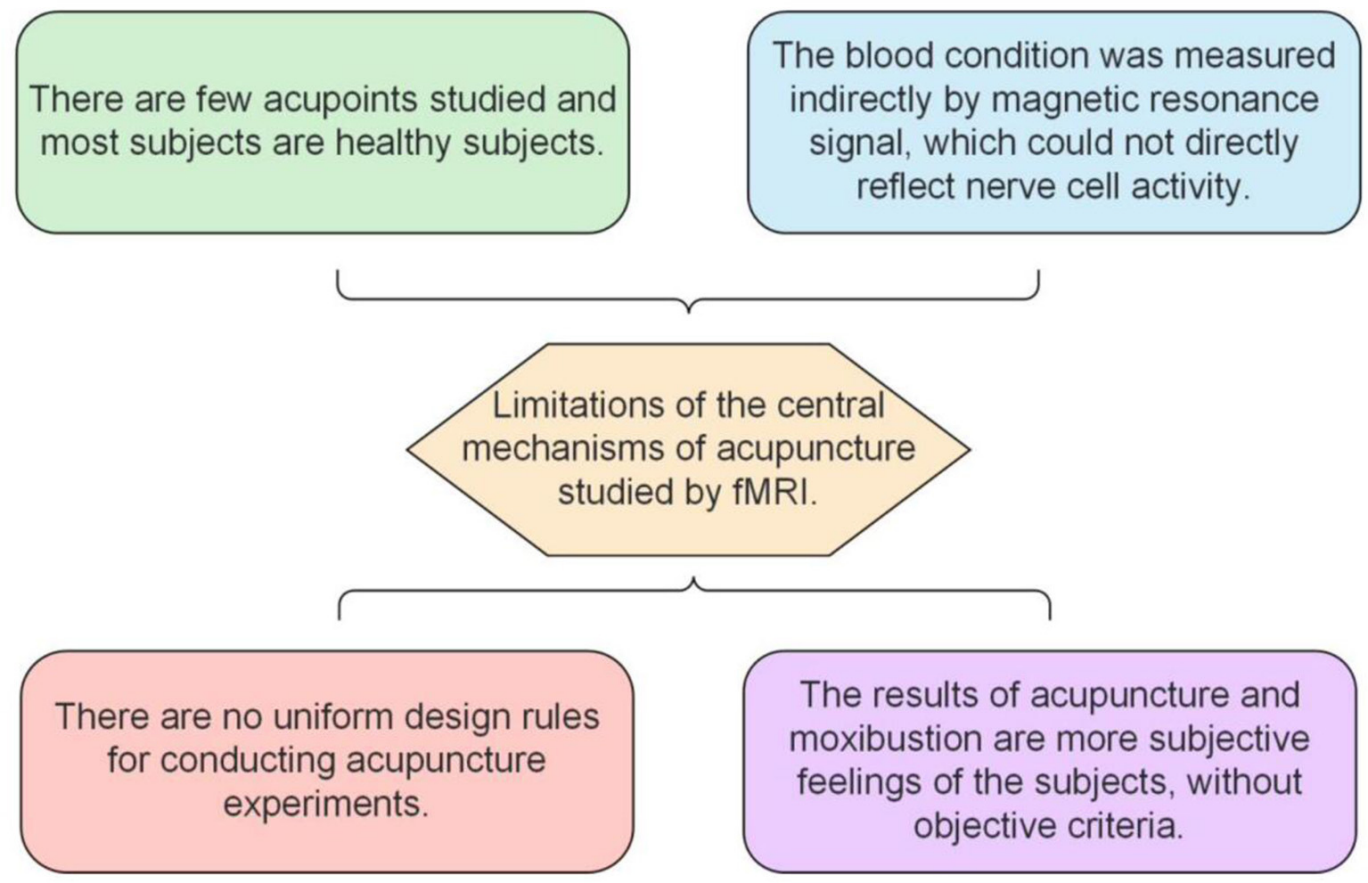
The effects of acupuncture and moxibustion, as observed by fMRI.

Although there are some shortcomings in the fMRI study of acupuncture, with the continuous improvement and development of fMRI, the study of the acupuncture mechanism can become more detailed. Figure 5 depicts the effects of acupuncture and moxibustion, as observed by fMRI.

## 6. Conclusion

fMRI study of the mechanism of the acupuncture effect on the brain is an emerging complex of functional imaging technology, computer science, brain science, and other interdisciplinary fields; it has greatly promoted relevant research on changes in the brain function of acupuncture acupoints and considerable progress has been made in the study of the acupuncture effect. fMRI research results can be usefully employed to study the real-time and immediate brain effect induced by acupuncture: activation and negative activation, different acupuncture methods, different acupoint stimulations to regulate specific brain function regions, stimulation effects of false acupoints, and acupuncture treatment of some diseases of the brain. Such study can help to guide the clinical practice of acupuncture and provide innovative research ideas for enhancing the employment of modern Chinese medicine.

It is a very challenging task to introduce fMRI technology into the study of acupuncture treatment of depression and to provide an fMRI image basis for the brain functional network. At present, fMRI studies on acupuncture for depression are limited to single or several independent brain functional networks related to depression, but they lack observation of the integration effect among brain functional networks. On the contrary, research results on the mechanism are not entirely systematic, and there is a need for a more scientific and systematic study of the fMRI-based “brain functional network mechanism of acupuncture” in the treatment of depression.

Depression affects a large number of brain regions. A large number of animal experiments and autopsies have shown that the amygdala and the dorsal raphe nucleus are closely related to depression. However, there are few relevant studies that observe the changes of the amygdala and the dorsal raphe nucleus in patients with depression before and after acupuncture treatment using fMRI. Compared with normal people, the nuclear mass of the cortex (such as the cingulate gyrus) shows significant changes in people with depression under fMRI. However, in terms of anatomical structure, the amygdala and the dorsal raphe nucleus are deeper than the cortex, so fMRI technology may not be able to detect the state of deep nuclei, for which there are few relevant studies. In future studies, if the limitation of nucleus mass location can be overcome by technological innovations to realize the observation of changes in brain nucleus masses, this will be conducive to improving the understanding of the central mechanism of depression, providing more valuable evidence for clinical practice. In addition, there are fiber connections in various brain regions, and the functional connections between brain regions can also be observed by fMRI technology. However, abnormal connections between brain regions are related to the incidence and severity of depression. Therefore, further research is needed on the neural circuits through which acupuncture can treat depression.

## Author contributions

Conceptualization: KW and JX. Investigation: CC and DG. Methodology: HZ, KW, and JX. Project administration and writing-original draft: JX and HZ. Visualization: KW and CC. Writing-review and editing: KW and HZ. All authors contributed to the article and approved the submitted version.
